# Supplementary material for: A highly potent and safe pyrrolopyridine-based allosteric HIV-1 integrase inhibitor targeting host LEDGF/p75-integrase interaction site
Source: PLoS Pathog. 2021 Jul 22;17(7):e1009671. doi: 10.1371/journal.ppat.1009671 (PMC8297771; doi:10.1371/journal.ppat.1009671)
Supplement: S1 Table — (PPTX) [file ppat.1009671.s001.pptx]

## Slide 1
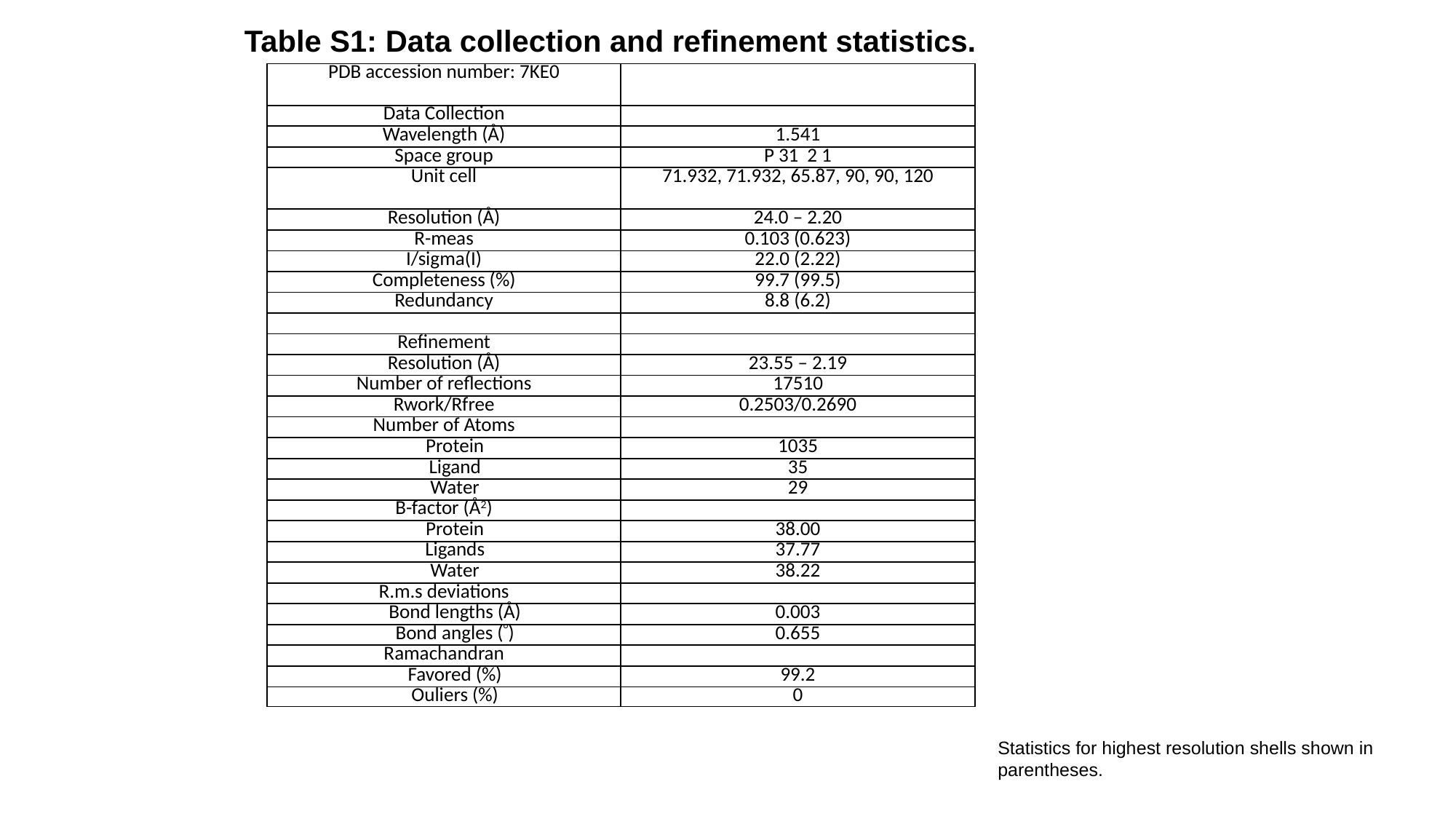

Table S1: Data collection and refinement statistics.
| PDB accession number: 7KE0 | |
| --- | --- |
| Data Collection | |
| Wavelength (Å) | 1.541 |
| Space group | P 31 2 1 |
| Unit cell | 71.932, 71.932, 65.87, 90, 90, 120 |
| Resolution (Å) | 24.0 – 2.20 |
| R-meas | 0.103 (0.623) |
| I/sigma(I) | 22.0 (2.22) |
| Completeness (%) | 99.7 (99.5) |
| Redundancy | 8.8 (6.2) |
| | |
| Refinement | |
| Resolution (Å) | 23.55 – 2.19 |
| Number of reflections | 17510 |
| Rwork/Rfree | 0.2503/0.2690 |
| Number of Atoms | |
| Protein | 1035 |
| Ligand | 35 |
| Water | 29 |
| B-factor (Å2) | |
| Protein | 38.00 |
| Ligands | 37.77 |
| Water | 38.22 |
| R.m.s deviations | |
| Bond lengths (Å) | 0.003 |
| Bond angles () | 0.655 |
| Ramachandran | |
| Favored (%) | 99.2 |
| Ouliers (%) | 0 |
Statistics for highest resolution shells shown in parentheses.
