# Supplementary material for: A highly potent and safe pyrrolopyridine-based allosteric HIV-1 integrase inhibitor targeting host LEDGF/p75-integrase interaction site
Source: PLoS Pathog. 2021 Jul 22;17(7):e1009671. doi: 10.1371/journal.ppat.1009671 (PMC8297771; doi:10.1371/journal.ppat.1009671)
Supplement: S2 Table — (PPTX) [file ppat.1009671.s002.pptx]

## Slide 1
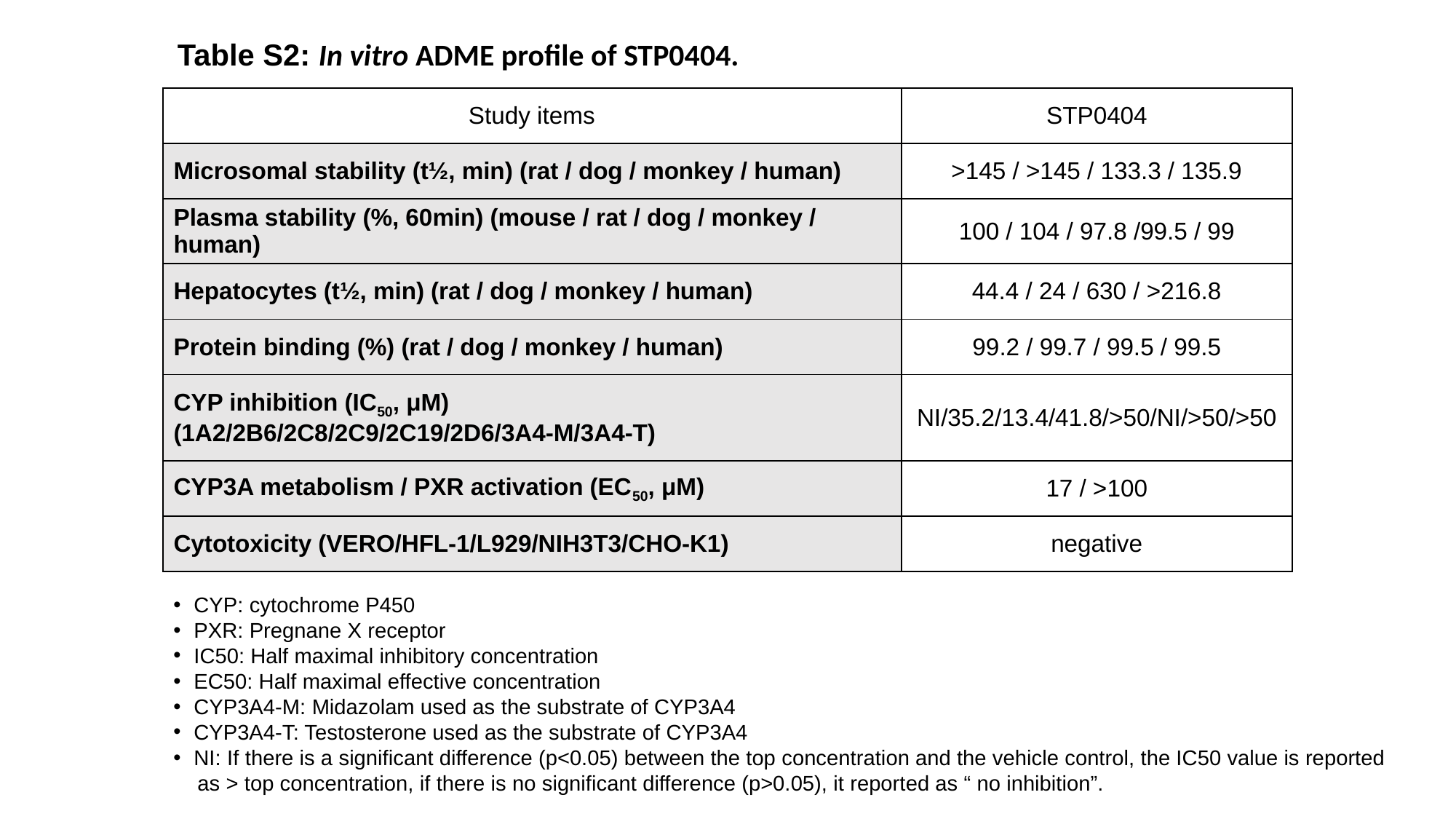

Table S2: In vitro ADME profile of STP0404.
| Study items | STP0404 |
| --- | --- |
| Microsomal stability (t½, min) (rat / dog / monkey / human) | >145 / >145 / 133.3 / 135.9 |
| Plasma stability (%, 60min) (mouse / rat / dog / monkey / human) | 100 / 104 / 97.8 /99.5 / 99 |
| Hepatocytes (t½, min) (rat / dog / monkey / human) | 44.4 / 24 / 630 / >216.8 |
| Protein binding (%) (rat / dog / monkey / human) | 99.2 / 99.7 / 99.5 / 99.5 |
| CYP inhibition (IC50, μM) (1A2/2B6/2C8/2C9/2C19/2D6/3A4-M/3A4-T) | NI/35.2/13.4/41.8/>50/NI/>50/>50 |
| CYP3A metabolism / PXR activation (EC50, μM) | 17 / >100 |
| Cytotoxicity (VERO/HFL-1/L929/NIH3T3/CHO-K1) | negative |
CYP: cytochrome P450
PXR: Pregnane X receptor
IC50: Half maximal inhibitory concentration
EC50: Half maximal effective concentration
CYP3A4-M: Midazolam used as the substrate of CYP3A4
CYP3A4-T: Testosterone used as the substrate of CYP3A4
NI: If there is a significant difference (p<0.05) between the top concentration and the vehicle control, the IC50 value is reported
 as > top concentration, if there is no significant difference (p>0.05), it reported as “ no inhibition”.
