# Supplementary material for: A highly potent and safe pyrrolopyridine-based allosteric HIV-1 integrase inhibitor targeting host LEDGF/p75-integrase interaction site
Source: PLoS Pathog. 2021 Jul 22;17(7):e1009671. doi: 10.1371/journal.ppat.1009671 (PMC8297771; doi:10.1371/journal.ppat.1009671)
Supplement: S3 Table — (PPTX) [file ppat.1009671.s003.pptx]

## Slide 1
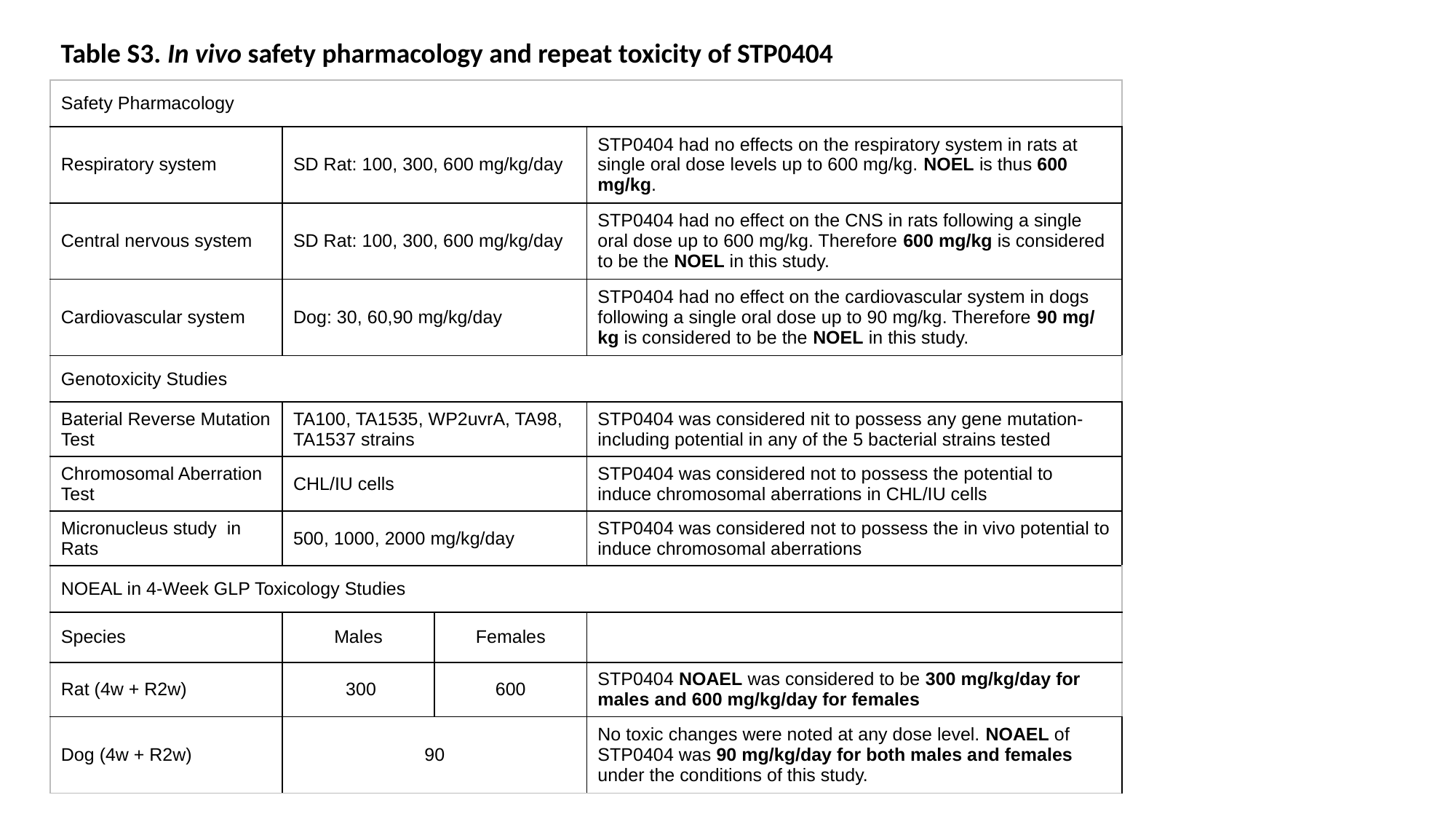

Table S3. In vivo safety pharmacology and repeat toxicity of STP0404
| Safety Pharmacology | | | |
| --- | --- | --- | --- |
| Respiratory system | SD Rat: 100, 300, 600 mg/kg/day | | STP0404 had no effects on the respiratory system in rats at single oral dose levels up to 600 mg/kg. NOEL is thus 600 mg/kg. |
| Central nervous system | SD Rat: 100, 300, 600 mg/kg/day | | STP0404 had no effect on the CNS in rats following a single oral dose up to 600 mg/kg. Therefore 600 mg/kg is considered to be the NOEL in this study. |
| Cardiovascular system | Dog: 30, 60,90 mg/kg/day | | STP0404 had no effect on the cardiovascular system in dogs following a single oral dose up to 90 mg/kg. Therefore 90 mg/kg is considered to be the NOEL in this study. |
| Genotoxicity Studies | | | |
| Baterial Reverse Mutation Test | TA100, TA1535, WP2uvrA, TA98, TA1537 strains | | STP0404 was considered nit to possess any gene mutation-including potential in any of the 5 bacterial strains tested |
| Chromosomal Aberration Test | CHL/IU cells | | STP0404 was considered not to possess the potential to induce chromosomal aberrations in CHL/IU cells |
| Micronucleus study in Rats | 500, 1000, 2000 mg/kg/day | | STP0404 was considered not to possess the in vivo potential to induce chromosomal aberrations |
| NOEAL in 4-Week GLP Toxicology Studies | | | |
| Species | Males | Females | |
| Rat (4w + R2w) | 300 | 600 | STP0404 NOAEL was considered to be 300 mg/kg/day for males and 600 mg/kg/day for females |
| Dog (4w + R2w) | 90 | | No toxic changes were noted at any dose level. NOAEL of STP0404 was 90 mg/kg/day for both males and females under the conditions of this study. |
