# Supplementary figures and images for: A highly potent and safe pyrrolopyridine-based allosteric HIV-1 integrase inhibitor targeting host LEDGF/p75-integrase interaction site
Source: PLoS Pathog. 2021 Jul 22;17(7):e1009671. doi: 10.1371/journal.ppat.1009671 (PMC8297771; doi:10.1371/journal.ppat.1009671)

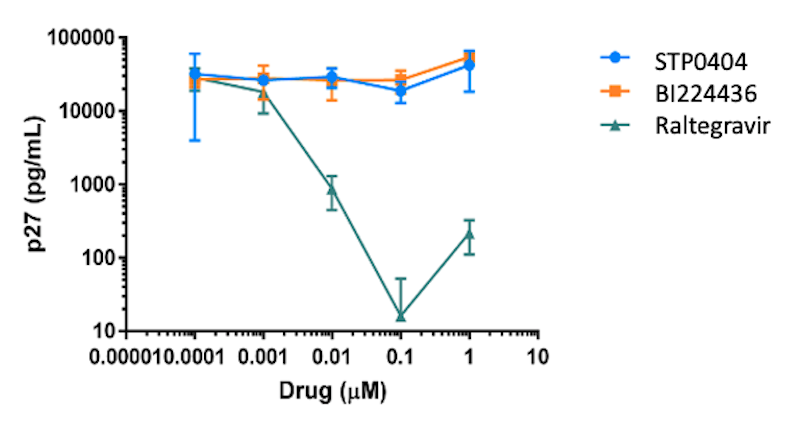

Supplement: S1 Fig — CEMx174 cells were infected with SIVmac239 with various concentrations of STP0404, BI224436, and Raltegravir, and the viral production was determined by p27 assay at 5 days post infection. (TIFF) [file ppat.1009671.s004.tiff]

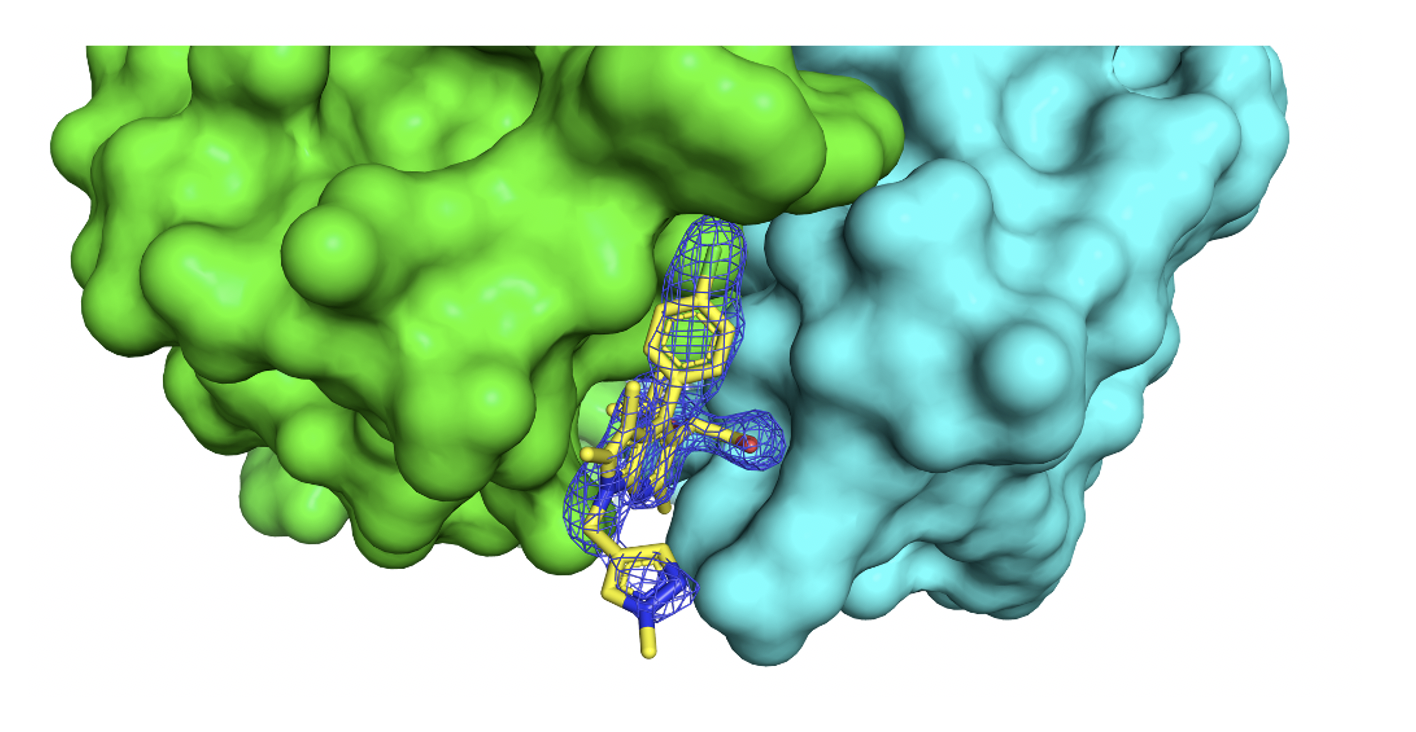

Supplement: S2 Fig — This is a close up image of the STP0404 binding (mesh) to the LEDGF/p75 binding pocket of IN dimer shown in Fig 3D. (TIFF) [file ppat.1009671.s005.tiff]
