# Supplementary material for: A highly potent and safe pyrrolopyridine-based allosteric HIV-1 integrase inhibitor targeting host LEDGF/p75-integrase interaction site
Source: PLoS Pathog. 2021 Jul 22;17(7):e1009671. doi: 10.1371/journal.ppat.1009671 (PMC8297771; doi:10.1371/journal.ppat.1009671)
Supplement: Attachment — Submitted filename: STP0404 PP Response.docx [file ppat.1009671.s006.docx]

**Reviewer's Responses to Questions**

(Our responses are in **bold**, and the revised sections in the manuscript are marked in **yellow**.)

**Part II – Major Issues: Key Experiments Required for Acceptance**

Reviewer #1: No major issues

Reviewer #2: 1- Given the big reduction in fitness with the Y99H substitution, the authors should probably have passaged this virus further in the presence of STP0404 to gain full resistance. Alternatively they can clone in previously described resistance mutations and see whether they more potently restore virion infectivity in the absence/presence of the compound.

**Response: We appreciate this suggestion. As suggested by the reviewer, we cultured the cloned Y99H virus for additional three viral passages with and without STP0404, and we sequenced multiple IN genes cloned from passage 3. We did not observe any additional mutations in these cloned IN genes and Y99H mutation remained unchanged. Therefore, it is unlikely that the Y99H mutant virus improved its fitness by gaining additional IN mutations at least during these three additional passages. We discussed this at the Discussion section of the revised manuscript (page 12, second paragraph).**

2- For the in vitro PK assays, I would love to see a comparison with 1-2 most potent ALLINIs. It is not feasible to repeat the entire in vitro PK studies.

**Response: Thank you for this suggestion. It is difficult to directly compare the PK profiles between STP0404 and other cases because different animal species and strains were used (BI224436 and GS9822): we added a discussion about this point with proper references (page 13, second paragraph).**

3- The in vivo PK studies are great but they fail to address one point which is whether the compound is effective against reducing virus titers in vivo. I think it would be important to show this in a humanized mouse model if at all possible.

**Response: While testing with mouse models would be informative for the in vivo efficacy, this compound has been already moved to human studies, which will confirm the true human in vivo efficacy. In addition, efficacy tests in animal models are not required for the IND filing for human trials of anti-HIV drugs.**

Reviewer #3: None

**Part III – Minor Issues: Editorial and Data Presentation Modifications**

Please use this section for editorial suggestions as well as relatively minor modifications of existing data that would enhance clarity.

 Reviewer #1: 1) Figure 4b: How many virions were imaged? Methods (page 16) should include at least very brief details of the staining and imaging procedures.

**Response: We captured images for ~1,000 virions, and this was described in the revised method section (page 17, second paragraph)**

2) Inhibition of IN-LEDGF interaction (page 8): I suggest highlighting that the assay used full-length HIV-1 IN. It is impressive that the small molecule can do that.

**Response: We revised as suggested (page 9, second paragraph).**

3) Is the compound active (or is not expected to be active) against HIV-2? Can we expect issues with some HIV-1 clades? I am not suggesting adding data; a few words on this would improve Discussion.

**Response: Instead of HIV-2, we actually tested SIVmac293 and STP0404 did not inhibit SIVmac293 (Figure S1). The sequences of HIV-2 (i.e. Rod) and SIV (i.e. SIVmac293) are significantly different from those of HIV-1. For example, both HIV-2 and SIVmac293 IN proteins encode Leu at residue 99 (Y99 in HIV-1) and Met at residue 128 (A128 in HIV-1), which explains the no efficacy of STP0404 against SIVmac239. This was discussed in the revised manuscript (page 13, first paragraph).**

4) An image of electron density of the drug-bound pocket should be included.

**Response: Figure 3d was revised as suggested and a close up image with STP0404 (mesh) was added as a new Supplementary figure (Figure S2).**

Reviewer #2: Perhaps intro and discussion can be expanded a little bit to compare the compound to other ALLINIs and why the authors think it is better.

**Response: STP0404 displays a high potency, no animal toxicity, and oral once-daily PK profile, which enabled us to move forward for FIH trials, and this description was added (the last sentence of Introduction).**

Reviewer #3: 1. "Low nanomolar" would appear to be a more accurate description of the IC50 range than "picomolar".

**Response: “Picomolar” IC50 values were originally used only when the tests were done with PBMCs specifically (e,g, page 5, first paragraph and abstract), and when the IC50 range was described (e.g. page 5, the last sentence of the first paragraph).**

2. While the data in Table 1 support the authors claim that STP0404 is highly potent in both wild-type and Ral-resistant HIV-1 strains, the reader is left wondering why the STP0404 IC50s are considerably lower in most of the Ral-resistant strains. Is this simply due to other differences in these strains compared to the Nl4-3 comparator? Some comment would be helpful.

**Response: It is correct that some clinical isolates appeared to be more sensitive to STP0404. We believe that those clinical isolates have different and diverse backgrounds that may influence the sensitivity to the compound.**
